# Prevalence and Adverse Outcomes of Iron Deficiency in Heart Failure

**DOI:** 10.2174/011573403X351268250130070459

**Published:** 2025-02-14

**Authors:** Habeeb Abdulkareem Habeeb, Fraser Todd, Rohith Valsalan, Emily Schembri, John K. Noyhar, Gary Yip, Mahesan Anpalahan

**Affiliations:** 1 Department of General Medicine, Eastern Health, Melbourne, Australia;; 2 Department of General Medicine, Eastern Health Clinical School, Monah University, Melbourne, Australia;; 3 Eastern Health Clinical School, Monash University, Melbourne, Australia

**Keywords:** Heart failure, iron deficiency, anaemia, mortality, readmission, HFrEF, HfpEF

## Abstract

**Introduction/Objective:**

Outcomes of iron deficiency (ID) in heart failure (HF) with preserved ejection fraction (HFpEF) or in samples with mixed heart failure subtypes are variably described. Hence, we investigated the prevalence and adverse outcomes of ID in a sample of mixed heart failure subtypes.

**Methods:**

Adult patients admitted with HF over a six-month period were retrospectively studied. ID was defined as serum ferritin<100 mcg/L, or serum ferritin 100-300 mcg/L with serum transferrin saturation<20%. For each case of ID, sex- and age-matched (within five years) controls were selected. The primary outcome was the composite of all-cause mortality or readmissions up to 12 months post-index admission.

**Results and Discussion:**

Of the 245 patients admitted with HF [HFpEF: 83 (70.3%), HFrEF: 35 (29.6%)], 233 met the inclusion criteria. Iron studies were available for 131 patients and 59 (45%) had ID. ID had a significant univariate association with the primary outcome (OR: 3.80, 95% CI: 1.42–10.18, *P=*0.008), and it remained significant after controlling for age, anaemia, comorbidities, and frailty (OR: 6.04, 95% CI: 1.18–30.85, *P=*0.031). ID also had a significant independent association with readmissions (OR: 4.61, 95% CI: 1.15–18.43, *P=*0.03), but not with mortality (OR: 1.17, 95% CI: 0.67-4.35, *P=*0.257). In post-hoc analysis, ID exhibited a significant association with primary outcome in patients with HFrEF (OR: 14.12, 95% CI: 1.7-117.33, *P=*0.014), but not in patients with HFpEF (OR: 1.8, 95% CI: 0.71-4.58, *P=*0.214).

**Conclusion:**

ID is common in patients hospitalised for heart failure and has been found to have a significant association with the composite primary outcome, largely due to its effect on readmissions. ID may have a differential effect on adverse outcomes with respect to heart failure subtypes.

## 1 INTRODUCTION

Heart failure is a leading cause of hospitalisation worldwide [1], with an overall prevalence between 1 and 2% in the Western world [2]. Studies have consistently shown that anaemia is highly prevalent in heart failure [3] and is associated with adverse outcomes, including mortality [4] and readmissions [5], in both heart failure with reduced ejection fraction (HFrEF) [3] and preserved ejection fraction (HFpEF) [6]. More recently, iron deficiency, a condition that is closely linked with anaemia, has emerged as a therapeutic target in heart failure. Although studies have investigated the prevalence and associated adverse outcomes of iron deficiency in heart failure, this has been found mostly in patients with HFrEF [7-11], and there is limited evidence in patients with HFpEF or in mixed samples consisting of patients with both HFrEF and HFpEF, especially in acute hospital settings. Outcomes in a mixed sample consisting of both types of heart failure are likely to be of greater practical significance because mixed samples are more reflective of real-world practice. Hence, this study was designed to investigate the prevalence of iron deficiency and the associated adverse outcomes of mortality and unplanned readmissions in a cohort of heart failure patients consisting of both HFrEF and HFpEF, who were admitted for decompensated heart failure.

## 2 MATERIALS AND METHODS

### 2.1 Study Design

A retrospective cohort study was performed where patients were followed up for 12 months after the index admission.

### 2.2 Setting

The study setting was a general internal medicine unit in a metropolitan teaching hospital in Melbourne, Australia.

### 2.3 Participants

All patients aged > 18 years admitted with a diagnosis of heart failure over a six-month period from 1^st^ February, 2018, to 31^st^ July, 2018, were eligible for inclusion in the study. The study was conducted in the years 2022/2023, but the participants were chosen from admissions in 2018 to avoid any potential impact of the COVID-19 pandemic on patients’ management and post-discharge follow-up. Patients were identified from the hospital health information database using the relevant ICD codes for heart failure (I11, I13, I25, I42, I43, I50, I51) and the diagnosis of heart failure was further verified by review of medical records. Patients were excluded if they were considered for palliative care and/or died within 24 hours of admission, had surgery, received a blood transfusion or iron infusion in the previous six months, received erythropoietic stimulating agents, or had active bleeding or concurrent haematological conditions. For patients who had multiple admissions during the study period, the first (index) admission was considered for analysis. Heart failure was classified on the basis of left ventricular ejection fraction (LVEF) measured on echocardiogram as HFrEF (LVEF <50%) and HFpEF (LVEF > 50%). Iron deficiency was defined as a serum ferritin level <100mcg/L (absolute iron deficiency) or a level 100-300mcg/L with a transferrin saturation (TSAT) level < 20 (functional iron deficiency), as per current heart failure guidelines [12]. Clinically significant anaemia (CSA) was arbitrarily defined by a serum Hb level < 110 g/l [13]. For each case of heart failure with iron deficiency, sex- and age-matched (within five years) control was randomly selected from patients admitted with heart failure without iron deficiency during the study period. The exclusion criteria for controls were the same as for cases. The study was approved by the Eastern Health Research Ethics Committee (Ref.: QA21-003) and performed in accordance with the principles of the Declaration of Helsinki of 1975 (and as revised in 2013).

### 2.4 Measurements and Data Collection

The relevant demographic, clinical, laboratory, and outcome data were extracted by review of electronic medical records using a structured data collection sheet. Patients’ general practitioners and outside providers were contacted when necessary. The data collected included age, gender, details of diagnosis on admission and comorbidities, baseline functional status, medications used, death during index admission or during 12 months of follow-up, and unplanned readmissions up to 12 months post-index admission. The laboratory data included haemoglobin, MCV, MCHC, iron levels, and estimated glomerular filtration rate (eGFR) and echocardiogram. The total burden of comorbidities was assessed by calculating the Charlson comorbidity index (CCI). The clinical frailty scale (CFS) was used to assess pre-admission baseline frailty.

### 2.5 Outcomes

The primary outcome was the composite of all-cause mortality or unplanned readmissions up to 12 months post-index admission. The secondary outcomes included individual components of the composite primary outcome, *i.e.*, 1) all-cause mortality during index admission or up to 12 months post-index admission, and 2) unplanned readmissions up to 12 months post-index admission.

### 2.6 Statistical Analysis

Descriptive information on sample characteristics was presented as an absolute number of cases and percentage of total group data or median (IQR). A univariate conditional logistic regression analysis was performed to determine the association between the predictor variables and outcomes of interest. A multivariate conditional logistic regression analysis was then undertaken to determine the unique contribution of iron deficiency to the primary and secondary outcomes after controlling for covariates. The covariates for the multivariate logistic regression were chosen based on their biological plausibility, including age, clinically significant anaemia, CCI, and CFS (>4 *vs.*<4), which were included *a priori* and/or considering the statistical association with the primary outcome in the univariate analysis, defined as a *P*-value of <0.1. Results were reported in adjusted odds ratios and 95% confidence intervals. A two-tailed *P* <0.05 indicated statistical significance. All analyses were performed using STATA v.18. Post-hoc analyses were undertaken to assess the effects of iron deficiency in HFpEF and HFrEF, using standard logistic regression. Additionally, post-hoc sensitivity analysis was also undertaken using a different threshold for CFS (> 3 *vs.* < 3) and including CCI as a categorical variable (CCI 2 ≤ *vs.* > 2) in order to assess the strength of the association between iron deficiency and primary composite outcome.

## 3 RESULTS

Of the 245 patients with heart failure admitted during the study period, 12 were excluded due to the following reasons: 4 of them died or were considered for palliative care within 24 hours of admission; 3 had a concomitant haematological condition; 5 had undergone surgery, blood transfusion, or iron infusion within six months of admission. Of the remaining 233 patients, iron studies were available for 131 of them, and 59 (45%) were found to have iron deficiency, 42 (71%) had absolute levels, and 17 (29%) had functional.

There were 59 heart failure patients with iron deficiency and an equal number of controls without iron deficiency. The baseline characteristics of the cases and controls are shown in Table **1**. The groups were similar with respect to their demographics, CFS, eGFR, and medication use. The majority of patients [83 (70.3%)] had heart failure with preserved ejection fraction, and they were evenly distributed between the groups. However, patients with iron deficiency were more likely to have significantly lower haemoglobin levels and suffer from clinically significant anaemia, and those without iron deficiency were more likely to have a higher CCI. As expected, a significantly higher proportion of patients in the iron deficiency group had biochemical and red cell indices consistent with iron deficiency.

**Fig. (1):**
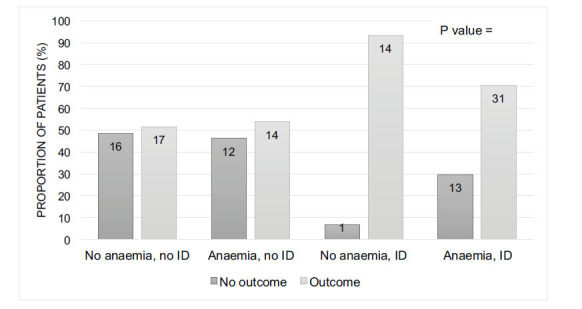
Proportion of patients with composite outcome, by clinically significant anaemia and iron deficiency status. † ID, Iron Deficiency.

The primary outcome, *i.e.*, the composite of all-cause mortality or unplanned readmissions up to 12 months, was found in 76 patients: 45 (76%) patients with iron deficiency and 31 (53%) without iron deficiency. The total number of deaths and unplanned readmissions occurred in 34 and 63 of the patients, respectively. The event rates of primary composite outcome in the groups of patients categorised based on the presence or absence of anaemia and iron deficiency were as follows: 1) patients without anaemia or iron deficiency (51.5%); 2) patients with anaemia, but no iron deficiency (53.8%); 3) patients with iron deficiency, but no anaemia (93.3%); and 4) patients with both anaemia and iron deficiency (70,4%) (*P=* 0.020) (Fig. **1**).

The results of the univariate analysis showing the association between the independent variables and the composite primary outcome are illustrated in Table **2**. As can be seen, iron deficiency had a significant univariate association with the primary outcome (OR 3.8: 95% CI: 1.42 – 10.18, *P=* 0.008), and this association remained significant after controlling for age, CSA, CCI, and CFS (OR: 6.04, 95% CI: 1.18 – 30.85, *P=*0.031) (Tables **2** and **3**). None of the other independent variables, including CCI, CFS, and CSA, had any significant association with the composite primary outcome (Table **2**). When breaking down the composite outcome into respective components, *i.e.*, unplanned readmissions and all-cause mortality, iron deficiency had a significant univariate association with unplanned readmissions (OR: 3.29, 95% CI: 1.41 – 7.66, *P* =0.006), and this association persisted when controlled for age, CSA, CCI, and CFS (OR: 4.61, 95% CI: 1.15 –1.43, *P=*0.031) (Table **3**). Iron deficiency, however, did not have a significant association with all-cause mortality, neither in the univariate (OR: 1.17, 95% CI: 0.67 – 4.35, *P=*0.257) nor multivariate model (OR: 1.91, 95% CI: 0.40 – 9.01, *P=*0.414) (Table **3**). Sensitivity analysis confirmed the results of the primary analysis, *i.e.*, the association between iron deficiency and the primary composite outcome did not change significantly from the primary model (Table **S1**).

The results of the post-hoc analysis revealed that ID did not have a significant association with the primary outcome (OR: 1.8, 95% CI: 0.71-.58, *P=*0.214) in those with HFpEF. However, it was significantly associated with the primary outcome in HFrEF (OR: 9.3, 95% CI: 1.91-45.58, *P=*0.006), and this association persisted when controlled for age, gender, CSA, CCI, and CFS (OR: 14.12, 95% CI: 1.7-0- 117.33, *P=*0.014).

## 4 DISCUSSION

The main findings of this study are that ID is common in patients hospitalised for decompensated heart failure and patients with ID are at a significantly greater risk for adverse outcomes, particularly unplanned readmissions, independently of age, gender, clinically significant anaemia, comorbidities, and frailty. The findings also suggest that ID may have a differential effect on adverse outcomes with respect to heart failure subtypes.

Unlike in HFrEF, only limited studies have described the prevalence or outcomes of ID in HFpEF or in mixed samples of heart failure subtypes, having a substantial number of patients with HFpEF. Núñez *et al*. reported a 74% prevalence of ID in a mixed sample of 693 patients with both heart failure subtypes. Similar to our study, their sample was derived from an inpatient setting and the majority of patients (52%) had HFpEF [14]. However, in most other studies involving mixed samples, there has been a significant under-representation of patients with HFpEF. Klip *et al*. reported a prevalence of 50% ID in a mixed sample of 1506 patients with stable chronic heart failure, but the majority of the sample comprised patients with HFrEF, and the mean EF of the sample was 33 +/- 14% [15]. Yeo *et al*. in a mixed sample of 751 patients of Southeast Asian origin with both heart failure subtypes, recruited from both outpatient and in-patient settings, reported a prevalence of 61.4% ID; however, only a minority of 20.5% of the sample had HFpEF [16]. Martens *et al*. reported a prevalence of 53% ID in a mixed sample of 1197 patients, but only 6.6% accounted for patients with HFpEF [17]. Therefore, the present study is an important advance in that the majority of the sample, over 70%, comprised of patients with HFpEF. The prevalence of iron deficiency observed in this study has been lower compared to other studies involving hospitalized patients with decompensated heart failure, irrespective of the heart failure subtype [18-20]. However, the prevalence observed in the present study should be interpreted with caution as iron studies were not available for all participants, thus potentially skewing the result. Additionally, the heterogeneity in sample characteristics, including heart failure subtypes, stable heart failure in outpatient settings versus decompensated heart failure in inpatient settings, along with differences in study design and definitions of iron deficiency, may complicate undertaking any meaningful comparisons between the studies.

This study confirmed the independent association between iron deficiency and the composite outcome of all-cause mortality or unplanned readmissions. This was driven primarily by readmissions rather than mortality. The association of iron deficiency with all-cause mortality [8], cardiovascular mortality [21], readmissions due to any cause [14], heart failure or other cardiovascular causes [22], and poor quality of life measures [11] has been demonstrated by many studies, but mostly among patients with HFrEF. As highlighted previously, most studies involving samples containing both subtypes of heart failure have not involved adequate representation of patients with HFpEF [15, 16]. Although a few outcome studies have included a sizeable number of patients with either HFpEF or mildly reduced ejection fraction (HFmrEF), only a few have investigated hard clinical outcomes. For example, Núñez *et al*. in a mixed sample, where 52% of patients had HFpEF, demonstrated 30-day readmission to be associated with ID, but the study outcomes did not include mortality or readmissions in the medium-to-long term [14]. The post-hoc analysis of a mixed sample by Josep Comin-Colet *et al*. indicated 48% of patients with LVEF >45%, but the study reported only outcomes of quality-of-life measures and not readmissions or mortality [23]. Graham *et al*., on the other hand, demonstrated increased mortality to be associated with ID in a sample of mixed heart failure subtypes. However, only 33% of the participants were included in the final analysis, and the majority (74%) of those excluded had either HFpEF or HFmrEF, and the exact number of patients with HFpEF or HFmrEF who completed the study was unclear [24]. Therefore, the present study is among the few to provide insights into hard clinical outcomes associated with ID over at least medium-term follow-up in a mixed sample consisting largely of patients with HFpEF.

The lack of association between iron deficiency and mortality in the present study may be due to the limited statistical power and shorter follow-up period, but, on the other hand, this aligns with other adequately powered studies that have failed to demonstrate any association with mortality [20, 25]. Furthermore, it is worth noting that RCTs [26-28] and meta-analyses [29-31] investigating the effects of intravenous iron therapy in patients with HFrEF and iron deficiency have also failed to confirm any mortality benefit and the benefits reported have been limited to readmissions and quality of life measures. Given the retrospective study design, the quality of life measures could not be assessed in the present study.

As in previous studies [32], the findings of the present study have also confirmed the association between iron deficiency and adverse outcomes, independent of anaemia. The findings of the univariate and multivariate analyses have suggested that iron deficiency may have a greater prognostic significance compared to anaemia. This has been further evidenced by the observation of the event rates of the primary outcome to be higher in patient groups with iron deficiency, with or without anaemia, compared to those with anaemia but no iron deficiency. This finding is broadly consistent with other studies that have compared adverse events between similar groups [10, 15]. However, it should be interpreted in the context of the small patient numbers in these groups (Fig. **1**).

The findings of the post-hoc analysis suggested that ID may have a differential effect with respect to heart failure subtypes and that ID may play a greater role in mediating adverse outcomes in HFrEF. The evidence for the impact of ID on clinical outcomes in HFpEF or HFmrEF is limited and inconsistent. Koseoglu and Ozlek, in a retrospective study of 212 heart failure patients with preserved ejection fraction and mean LVEF 57 ± 4.2%, in an outpatient setting, found that both anaemia and ID independently predicted all-cause mortality at a follow-up of 5.5 years [33]. Hospitalisation was not an outcome in this study. Aizpurua *et al*., in a prospective study of 300 patients with HFpEF, demonstrated that ID significantly correlated with increased all-cause mortality at a median follow-up of 47 months, but not with hospitalisation [34]. On the other hand, consistent with the findings of the post-hoc analysis of this study, Fitzsimons *et al*., in a mixed sample of heart failure subtypes, demonstrated iron deficiency to not be predictive of either all-cause mortality or the combined end point of death or heart failure hospitalization in patients with HFpEF. They further demonstrated that ID, defined based on transferrin saturation, predicted both outcomes in the whole sample as well as in patients with HFrEF [35]. Beale *et al*., in a systematic review and meta-analysis, concluded that while ID was associated with poor exercise capacity and functional outcomes in HFpEF, it was not associated with hospitalisation or mortality [20]. José González-Costello *et al*., in a mixed sample of heart failure subtypes with stable heart failure, where HFpEF accounted for 25% of the sample, found no association between ID and mortality or hospital admissions [26]. The authors hypothesised that the negative results could be due to the presence of a high proportion of patients with HFpEF in the sample. Although the findings of the present study may support this hypothesis and the conclusions of the systematic review and meta-analysis by Beale *et al*. [20], they require prospective evaluation in an adequately powered sample. The results of the FAIR-HFpEF trial, an RCT investigating the efficacy of intravenous ferric carboxymaltose in HFpEF that is currently underway, may provide some answers to the current knowledge gaps in HFpEF/HFmrEF. However, the study is unlikely to shed any light on outcomes, such as readmissions or mortality, as the primary objective of this study was to explore outcomes, such as exercise capacity and health-related quality of life measures [36].

## 5 STRENGTHS AND LIMITATIONS

The results of this study should be interpreted within the context of potential strengths and weaknesses. The sample size was small, and the study lacked any formal power calculation as it was a time-based study; however, post-hoc power calculation confirmed the results to be 94.8% valid. We believe the multivariate model including all the important confounding variables relevant to readmission, such as age, frailty, comorbidities, and anaemia, to be an important strength of this study. Additionally, the robustness of the association between iron deficiency and primary outcome was further established by the sensitivity analysis. The study suffered from the usual limitations of a retrospective study design, including selection, information, and classification biases. However, we believe that these biases would have operated equally among patients with and without iron deficiency as they were sampled contemporaneously. Given the small sample size and low number of event rates, the effects of ID on cardiac-specific outcomes could not be assessed. The study also did not account for patients who might have been readmitted to hospitals outside of Eastern Health. However, this number is likely negligible, as Eastern Health is the major healthcare provider in the region, with three acute sites. Given the lack of statistical power and wide confidence intervals, the results of the subgroup analysis should only be considered hypothesis-generating rather than confirmatory.

## CONCLUSION

The findings of this real-world study have confirmed that in a mixed sample of decompensated heart failure patients, with a high proportion of HFpEF, iron deficiency is common and significantly associated with readmissions, independent of anaemia, comorbidities, frailty, gender, and age. The study has also provided further insights into adverse outcomes of iron deficiency in HFpEF; however, these findings need prospective confirmation in an adequately powered study.

## Figures and Tables

**Table 1:** Baseline characteristics of patients with and without iron deficiency.

**Characteristics**	**Total**	**Patients with Iron Deficiency**	**Patients without Iron Deficiency**	***P*-value**
Age - Median (IQR) in years	87(11)	87(11.5)	87(10)	0.882
Sex	60Males-58 Females	30 Male-29 Female	30 Male-29 Female	0.999
CCI- Median (IQR)	6(2)	6(2.5)	6(3)	0.022
CFS≥4-Number (Percentage)	107(90.6%)	56(94.9%)	51(86.4%)	0.204
**Medications**				
Beta Blockers – Number (Percentage)	54(45.76%)	23 (38.98%)	31(52.54%)	0.139
ACEI/ARBs- Number (Percentage)	48(40.68%)	21 (35.59%)	27(45.76%)	0.261
Diuretics- Number (Percentage)	102(86.44%)	54(91.53%)	48(81.36%)	0.107
Antiplatelets- Number (Percentage)	50(42.37%)	27(45.76%)	23(38.98%)	0.456
Anticoagulants- Number (Percentage)	47(39.83%)	26(44.07%)	21(35.59%)	0.347
**Investigation Findings**				
Clinically significant anaemia-Number (Percentage)	70(59.32%)	44(74.58%)	26(44.07%)	0.001
Hb†-Median (IQR)	106.5(21.75)	100(19.5)	114(27)	<0.001
MCHC‡-Median (IQR)	325.5(13.75)	323(19)	329(15.5)	0.004
MCV§- Median (IQR)	88(13)	82(11)	92(10.5)	<0.001
TSAT¶- Median (IQR)	17(19.5)	10(9.5)	27(15.5)	<0.001
Ferritin÷- Median (IQR)	222(369.5)	64(67)	435 (354.5)	<0.001
eGFRµ - Median (IQR)	40(29.5)	42(29.5)	39 (28.5)	0.225
HFpEFℬ - Number (Percentage)	83(70.33%)	42(71.1%)	41(69.49%)	0.840

† Hb, Haemoglobin.

‡ MCHC, Mean corpuscular haemoglobin concentration.

§ MCV, Mean corpuscular volume.

¶TSAT, Transferrin saturation.

÷ACEI/ARB, Angiotensin converting enzyme inhibitors and angiotensin receptor blockers.

µ eGFR, Estimated Glomerular filtration rate.

ℬ HFpEF, Heart failure with preserved ejection fraction.

**Table 2:** Results of univariate regression analysis.

**Composite Outcome**			
**Variable**	**OR**	**95% CI**	***P*-value**
Iron deficiency	3.8	1.42 – 10.18	0.008
Age	1.00	0.76 – 1.32	0.999
CCI†	1.33	0.97 – 1.81	0.073
CFS‡	2.00	0.18 – 22.06	0.571
CSA§	1.25	0.34 – 4.65	0.739
HFpEF¶	0.67	0.19 – 2.36	0.530
eGFRµ	1.01	0.98 – 1.04	0.539
Beta-Blockers	0.86	0.29 – 2.55	0.782
ACEI/ARBs-÷	1.67	0.39 – 3.47	0.782
Diuretics	2.5	0.49 – 12.89	0.273
Antiplatelet	0.86	0.29 – 2.55	0.782
Anticoagulants	1.40	0.44 – 4.41	0.566
Hb ℬ	0.96	0.92 – 1.01	0.135
MCHC %	0.98	0.94 – 1.02	0.246
MCV &	0.95	0.87 – 1.04	0.232
TSTAT !	0.98	0.94 – 1.03	0.470
Ferritin	0.99	0.99 – 1.00	0.108

† CCI, Charlson Comorbidity Index.

‡ CFS, Clinical Frailty Scale.

§ CSA, Clinically Significant Anaemia.

¶ HFpEF, Heart failure with preserved ejection fraction.

÷ACEI/ARB, Angiotensin converting enzyme inhibitors and angiotensin receptor blockers.

µ eGFR, Estimated Glomerular filtration rate.

ℬ Hb, Haemoglobin.

% MCHC, Mean corpuscular haemoglobin concentration.

& MCV, Mean corpuscular volume.

! TSAT, Transferrin Saturation.

**Table 3:** Results of multivariate regression analysis.

**Variable**	**OR**	**95% CI**	***P*-value**
**Composite Outcome**			
Iron deficiency	6.04	1.18 – 30.85	0.031
Age	1.04	0.69 – 1.55	0.862
CCI†	1.23	0.76 – 1.98	0.407
CFS‡	0.27	0.01 – 6.48	0.420
CSA§	0.19	0.02 – 1.65	0.132
**Readmission**			
Fe deficiency	4.61	1.15 – 18.43	0.031
Age	1.00	0.69 – 1.49	0.988
CCI†	1.49	0.94 – 2.35	0.084
CFS‡	0.18	0.01 -4.77	0.304
CSA§	0.17	0.02 – 1.29	0.086
**Mortality**			
Iron deficiency	1.91	0.40 – 9.01	0.414
Age	0.95	0.55 – 1.63	0.839
CCI†	1.19	0.86 – 1.66	0.292
CFS‡	4.95	0.20 – 120.71	0.327
CSA§	0.28	0.03 – 2.56	0.259

† CCI, Charlson Comorbidity Index.

‡ CFS, Clinical Frailty Scale.

§ CSA, Clinically Significant Anaemia.

## Data Availability

The data that support the findings of the study are available from the authors, upon request, after obtaining approval from the Eastern Health Human Research and Ethics Committee.

## LIST OF ABBREVIATIONS

HF: Heart Failure
ID: Iron Deficiency
CFS: Clinical Frailty Scale
CCI: Charlson Comorbidity Index

## STANDARDS OF REPORTING

STROBE guidelines were followed.

## References

[1] Shahim B., Kapelios C.J., Savarese G., Lund L.H. (2023). Global public health burden of heart failure: An updated review.. Card. Fail. Rev..

[2] Groenewegen A., Rutten F.H., Mosterd A., Hoes A.W. (2020). Epidemiology of heart failure.. Eur. J. Heart Fail..

[3] Anand I.S., Gupta P. (2018). Anemia and iron deficiency in heart failure.. Circulation.

[4] Groenveld H.F., Januzzi J.L., Damman K., van Wijngaarden J., Hillege H.L., van Veldhuisen D.J., van der Meer P. (2008). Anemia and mortality in heart failure patients a systematic review and meta-analysis.. J. Am. Coll. Cardiol..

[5] Komajda M., Anker S.D., Charlesworth A., Okonko D., Metra M., Di Lenarda A., Remme W., Moullet C., Swedberg K., Cleland J.G.F., Poole-Wilson P.A. (2006). The impact of new onset anaemia on morbidity and mortality in chronic heart failure: Results from COMET.. Eur. Heart J..

[6] Majmundar M., Doshi R., Zala H., Shah P., Adalja D., Shariff M., Kumar A. (2021). Prognostic role of anemia in heart failure with preserved ejection fraction: A systematic review and meta-analysis.. Indian Heart J..

[7] von Haehling S., Gremmler U., Krumm M., Mibach F., Schön N., Taggeselle J., Dahm J.B., Angermann C.E. (2017). Prevalence and clinical impact of iron deficiency and anaemia among outpatients with chronic heart failure: The PrEP registry.. Clin. Res. Cardiol..

[8] Jankowska E.A., Rozentryt P., Witkowska A., Nowak J., Hartmann O., Ponikowska B., Borodulin-Nadzieja L., Banasiak W., Polonski L., Filippatos G., McMurray J.J.V., Anker S.D., Ponikowski P. (2010). Iron deficiency: An ominous sign in patients with systolic chronic heart failure.. Eur. Heart J..

[9] Jankowska E.A., Rozentryt P., Witkowska A., Nowak J., Hartmann O., Ponikowska B., Borodulin-Nadzieja L., von Haehling S., Doehner W., Banasiak W., Polonski L., Filippatos G., Anker S.D., Ponikowski P. (2011). Iron deficiency predicts impaired exercise capacity in patients with systolic chronic heart failure.. J. Card. Fail..

[10] Okonko D.O., Mandal A.K.J., Missouris C.G., Poole-Wilson P.A. (2011). Disordered iron homeostasis in chronic heart failure: Prevalence, predictors, and relation to anemia, exercise capacity, and survival.. J. Am. Coll. Cardiol..

[11] Enjuanes C., Klip I.J.T., Bruguera J., Cladellas M., Ponikowski P., Banasiak W., van Veldhuisen D.J., van der Meer P., Jankowska E.A., Comín-Colet J. (2014). Iron deficiency and health-related quality of life in chronic heart failure: Results from a multicenter European study.. Int. J. Cardiol..

[12] McDonagh T.A., Metra M., Adamo M., Gardner R.S., Baumbach A., Böhm M., Burri H., Butler J., Čelutkienė J., Chioncel O., Cleland J.G.F., Coats A.J.S., Crespo-Leiro M.G., Farmakis D., Gilard M., Heymans S., Hoes A.W., Jaarsma T., Jankowska E.A., Lainscak M., Lam C.S.P., Lyon A.R., McMurray J.J.V., Mebazaa A., Mindham R., Muneretto C., Francesco Piepoli M., Price S., Rosano G.M.C., Ruschitzka F., Kathrine Skibelund A., de Boer R.A., Christian Schulze P., Abdelhamid M., Aboyans V., Adamopoulos S., Anker S.D., Arbelo E., Asteggiano R., Bauersachs J., Bayes-Genis A., Borger M.A., Budts W., Cikes M., Damman K., Delgado V., Dendale P., Dilaveris P., Drexel H., Ezekowitz J., Falk V., Fauchier L., Filippatos G., Fraser A., Frey N., Gale C.P., Gustafsson F., Harris J., Iung B., Janssens S., Jessup M., Konradi A., Kotecha D., Lambrinou E., Lancellotti P., Landmesser U., Leclercq C., Lewis B.S., Leyva F., Linhart A., Løchen M-L., Lund L.H., Mancini D., Masip J., Milicic D., Mueller C., Nef H., Nielsen J-C., Neubeck L., Noutsias M., Petersen S.E., Sonia Petronio A., Ponikowski P., Prescott E., Rakisheva A., Richter D.J., Schlyakhto E., Seferovic P., Senni M., Sitges M., Sousa-Uva M., Tocchetti C.G., Touyz R.M., Tschoepe C., Waltenberger J., Adamo M., Baumbach A., Böhm M., Burri H., Čelutkienė J., Chioncel O., Cleland J.G.F., Coats A.J.S., Crespo-Leiro M.G., Farmakis D., Gardner R.S., Gilard M., Heymans S., Hoes A.W., Jaarsma T., Jankowska E.A., Lainscak M., Lam C.S.P., Lyon A.R., McMurray J.J.V., Mebazaa A., Mindham R., Muneretto C., Piepoli M.F., Price S., Rosano G.M.C., Ruschitzka F., Skibelund A.K. (2021). 2021 ESC Guidelines for the diagnosis and treatment of acute and chronic heart failure.. Eur. Heart J..

[13] Pisaniello A.D., Wong D.T.L., Kajani I., Robinson K., Shakib S. (2013). Anaemia in chronic heart failure: More awareness is required.. Intern. Med. J..

[14] Núñez J., Comín-Colet J., Miñana G., Núñez E., Santas E., Mollar A., Valero E., García-Blas S., Cardells I., Bodí V., Chorro F.J., Sanchis J. (2016). Iron deficiency and risk of early readmission following a hospitalization for acute heart failure.. Eur. J. Heart Fail..

[15] Klip I.J.T., Comin-Colet J., Voors A.A., Ponikowski P., Enjuanes C., Banasiak W., Lok D.J., Rosentryt P., Torrens A., Polonski L., van Veldhuisen D.J., van der Meer P., Jankowska E.A. (2013). Iron deficiency in chronic heart failure: An international pooled analysis.. Am. Heart J..

[16] Yeo T.J., Yeo P.S., Ching-Chiew Wong R., Ong H.Y., Leong K.T., Jaufeerally F., Sim D., Santhanakrishnan R., Lim S.L., M Y Chan M., Chai P., Low A.F., Ling L.H., Ng T.P., Richards A.M., Lam C.S. (2014). Iron deficiency in a multi-ethnic Asian population with and without heart failure: Prevalence, clinical correlates, functional significance and prognosis.. Eur. J. Heart Fail..

[17] Martens P., Nijst P., Verbrugge F.H., Smeets K., Dupont M., Mullens W. (2018). Impact of iron deficiency on exercise capacity and outcome in heart failure with reduced, mid-range and preserved ejection fraction.. Acta Cardiol..

[18] Cohen-Solal A., Damy T., Terbah M., Kerebel S., Baguet J.P., Hanon O., Zannad F., Laperche T., Leclercq C., Concas V., Duvillié L., Darné B., Anker S., Mebazaa A. (2014). High prevalence of iron deficiency in patients with acute decompensated heart failure.. Eur. J. Heart Fail..

[19] Nanas J.N., Matsouka C., Karageorgopoulos D., Leonti A., Tsolakis E., Drakos S.G., Tsagalou E.P., Maroulidis G.D., Alexopoulos G.P., Kanakakis J.E., Anastasiou-Nana M.I. (2006). Etiology of anemia in patients with advanced heart failure.. J. Am. Coll. Cardiol..

[20] Beale A.L., Warren J.L., Roberts N., Meyer P., Townsend N.P., Kaye D. (2019). Iron deficiency in heart failure with preserved ejection fraction: A systematic review and meta-analysis.. Open Heart.

[21] Cleland J.G.F., Zhang J., Pellicori P., Dicken B., Dierckx R., Shoaib A., Wong K., Rigby A., Goode K., Clark A.L. (2016). Prevalence and outcomes of anemia and hematinic deficiencies in patients with chronic heart failure.. JAMA Cardiol..

[22] Beattie J.M., Khatib R., Phillips C.J., Williams S.G. (2020). Iron deficiency in 78 805 people admitted with heart failure across England: A retrospective cohort study.. Open Heart.

[23] Comín-Colet J., Enjuanes C., González G., Torrens A., Cladellas M., Meroño O., Ribas N., Ruiz S., Gómez M., Verdú J.M., Bruguera J. (2013). Iron deficiency is a key determinant of health‐related quality of life in patients with chronic heart failure regardless of anaemia status.. Eur. J. Heart Fail..

[24] Graham F.J., Masini G., Pellicori P., Cleland J.G.F., Greenlaw N., Friday J., Kazmi S., Clark A.L. (2022). Natural history and prognostic significance of iron deficiency and anaemia in ambulatory patients with chronic heart failure.. Eur. J. Heart Fail..

[25] González-Costello J., Comín-Colet J., Lupón J., Enjuanes C., de Antonio M., Fuentes L., Moliner-Borja P., Farré N., Zamora E., Manito N., Pujol R., Bayés-Genis A. (2018). Importance of iron deficiency in patients with chronic heart failure as a predictor of mortality and hospitalizations: Insights from an observational cohort study.. BMC Cardiovasc. Disord..

[26] Ponikowski P., Kirwan B.A., Anker S.D., McDonagh T., Dorobantu M., Drozdz J., Fabien V., Filippatos G., Göhring U.M., Keren A., Khintibidze I., Kragten H., Martinez F.A., Metra M., Milicic D., Nicolau J.C., Ohlsson M., Parkhomenko A., Pascual-Figal D.A., Ruschitzka F., Sim D., Skouri H., van der Meer P., Lewis B.S., Comin-Colet J., von Haehling S., Cohen-Solal A., Danchin N., Doehner W., Dargie H.J., Motro M., Butler J., Friede T., Jensen K.H., Pocock S., Jankowska E.A., Azize G., Fernandez A., Zapata G.O., Garcia Pacho P., Glenny A., Ferre Pacora F., Parody M.L., Bono J., Beltrano C., Hershson A., Vita N., Luquez H.A., Cestari H.G., Fernandez H., Prado A., Berli M., García Durán R., Thierer J., Diez M., Lobo Marquez L., Borelli R.R., Hominal M.Á., Metra M., Ameri P., Agostoni P., Salvioni A., Fattore L., Gronda E., Ghio S., Turrini F., Uguccioni M., Di Biase M., Piepoli M., Savonitto S., Mortara A., Terrosu P., Fucili A., Boriani G., Midi P., Passamonti E., Cosmi F., van der Meer P., Van Bergen P., van de Wetering M., Al-Windy N.Y.Y., Tanis W., Meijs M., Groutars R.G.E.J., The H.K.S., Kietselaer B., van Kesteren H.A.M., Beelen D.P.W., Heymeriks J., Van de Wal R., Schaap J., Emans M., Westendorp P., Nierop P.R., Nijmeijer R., Manintveld O.C., Dorobantu M., Darabantiu D.A., Zdrenghea D., Toader D.M., Petrescu L., Militaru C., Crisu D., Tomescu M.C., Stanciulescu G., Rodica Dan A., Iosipescu L.C., Serban D.L., Drozdz J., Szachniewicz J., Bronisz M., Tycińska A., Wozakowska-Kaplon B., Mirek-Bryniarska E., Gruchała M., Nessler J., Straburzyńska-Migaj E., Mizia-Stec K., Szelemej R., Gil R., Gąsior M., Gotsman I., Halabi M., Shochat M., Shechter M., Witzling V., Zukermann R., Arbel Y., Flugelman M., Ben-Gal T., Zvi V., Kinany W., Weinstein J.M., Atar S., Goland S., Milicic D., Horvat D., Tušek S., Udovicic M., Šutalo K., Samodol A., Pesek K., Artuković M., Ružić A., Šikić J., McDonagh T., Trevelyan J., Wong Y-K., Gorog D., Ray R., Pettit S., Sharma S., Kabir A., Hamdan H., Tilling L., Baracioli L., Nigro Maia L., Dutra O., Reis G., Pimentel Filho P., Saraiva J.F., Kormann A., dos Santos F.R., Bodanese L., Almeida D., Precoma D., Rassi S., Costa F., Kabbani S., Abdelbaki K., Abdallah C., Arnaout M.S., Azar R., Chaaban S., Raed O., Kiwan G., Hassouna B., Bardaji A., Zamorano J., del Prado S., Gonzalez Juanatey J.R., Ga Bosa Ojeda F.I., Gomez Bueno M., Molina B.D., Pascual Figal D.A., Sim D., Yeo T.J., Loh S.Y., Soon D., Ohlsson M., Smith J.G., Gerward S., Khintibidze I., Lominadze Z., Chapidze G., Emukhvari N., Khabeishvili G., Chumburidze V., Paposhvili K., Shaburishvili T., Khabeishvili G., Parhomenko O., Kraiz I., Koval O., Zolotaikina V., Malynovsky Y., Vakaliuk I., Rudenko L., Tseluyko V., Stanislavchuk M. (2020). Ferric carboxymaltose for iron deficiency at discharge after acute heart failure: A multicentre, double-blind, randomised, controlled trial.. Lancet.

[27] Anker S.D., Comin Colet J., Filippatos G., Willenheimer R., Dickstein K., Drexler H., Lüscher T.F., Bart B., Banasiak W., Niegowska J., Kirwan B.A., Mori C., von Eisenhart Rothe B., Pocock S.J., Poole-Wilson P.A., Ponikowski P. (2009). Ferric carboxymaltose in patients with heart failure and iron deficiency.. N. Engl. J. Med..

[28] Ponikowski P., van Veldhuisen D.J., Comin-Colet J., Ertl G., Komajda M., Mareev V., McDonagh T., Parkhomenko A., Tavazzi L., Levesque V., Mori C., Roubert B., Filippatos G., Ruschitzka F., Anker S.D. (2015). Beneficial effects of long-term intravenous iron therapy with ferric carboxymaltose in patients with symptomatic heart failure and iron deficiency.. Eur. Heart J..

[29] Jankowska E.A., Tkaczyszyn M., Suchocki T., Drozd M., von Haehling S., Doehner W., Banasiak W., Filippatos G., Anker S.D., Ponikowski P. (2016). Effects of intravenous iron therapy in iron‐deficient patients with systolic heart failure: A meta‐analysis of randomized controlled trials.. Eur. J. Heart Fail..

[30] Khan M.S., Usman M.S., von Haehling S., Doehner W., Stewart Coats A.J. (2020). Ferric carboxymaltose for the treatment of iron‐deficient heart failure patients: A systematic review and meta‐analysis.. ESC Heart Fail..

[31] Anker S.D., Kirwan B.A., van Veldhuisen D.J., Filippatos G., Comin-Colet J., Ruschitzka F., Lüscher T.F., Arutyunov G.P., Motro M., Mori C., Roubert B., Pocock S.J., Ponikowski P. (2018). Effects of ferric carboxymaltose on hospitalisations and mortality rates in iron‐deficient heart failure patients: An individual patient data meta‐analysis.. Eur. J. Heart Fail..

[32] Rangel I., Gonçalves A., de Sousa C., Leite S., Campelo M., Martins E., Amorim S., Moura B., Silva Cardoso J., Maciel M.J. (2014). Iron deficiency status irrespective of anemia: A predictor of unfavorable outcome in chronic heart failure patients.. Cardiology.

[33] Köseoğlu F.D., Özlek B. (2024). Anemia and iron deficiency predict all-cause mortality in patients with heart failure and preserved ejection fraction: 6-year follow-up study.. Diagnostics.

[34] Barandiarán Aizpurua A., Sanders-van Wijk S., Brunner-La Rocca H.P., Henkens M.T.H.M., Weerts J., Spanjers M.H.A., Knackstedt C., van Empel V.P.M. (2021). Iron deficiency impacts prognosis but less exercise capacity in heart failure with preserved ejection fraction.. ESC Heart Fail..

[35] Fitzsimons S., Yeo T.J., Ling L.H., Sim D., Leong K.T.G., Yeo P.S.D., Ong H.Y., Jaufeerally F., Ng T.P., Poppe K., Lund M., Devlin G., Troughton R., Lam C.S.P., Richards A.M., Doughty R.N. (2021). Impact of change in iron status over time on clinical outcomes in heart failure according to ejection fraction phenotype.. ESC Heart Fail..

[36] von Haehling S., Doehner W., Evertz R., Garfias-Veitl T., Diek M., Karakas M., Birkemeyer R., Fillippatos G., Ponikowski P., Böhm M., Friede T., Anker S.D. (2023). Iron deficiency in heart failure with preserved ejection fraction: Rationale and design of the FAIR-HFpEF trial.. Global Cardiology.

