# Supplementary material for: Prevalence and Adverse Outcomes of Iron Deficiency in Heart Failure
Source: Curr Cardiol Rev. 2025 Feb 14;21(5):E1573403X351268. doi: 10.2174/011573403X351268250130070459 (PMC12307997; doi:10.2174/011573403X351268250130070459)
Supplement: Supplementary file 1 [file CCR-21-5-E1573403X351268_SD1.pdf]

Supplementary Material

Prevalence and Adverse Outcomes of Iron Deficiency in Heart Failure

Habeeb Abdulkareem Habeeb[1], Fraser Todd[1], Rohith Valsalan[2], Emily Schembri[3], John K. Noyhar[1], Gary Yip[2] and Mahesan Anpalahan[2,*]

[1]*Department of General Medicine, Eastern Health, Melbourne, Australia;* [2]*Department of General Medicine, Eastern Health Clinical School, Monah University, Melbourne, Australia;* [3]*Eastern Health Clinical School, Monash University, Melbourne, Australia*

Table S1: Results of post-hoc sensitivity analysis compared to original analysis.

Variable	Adjusted OR*	95% CI	P- value
**ID *vs* Composite**			
Original model	6.04	1.18 – 30.85	0.031
Sensitivity analysis model	5.25	1.18 – 23.25	0.029
**ID *vs* Readmission**			
Original model	4.61	1.15 – 18.43	0.03
Sensitivity analysis model	4.87	1.30 – 18.19	0.018
**ID *vs* Mortality**			
Original model	1.91	0.40 – 9.01	0.414
Sensitivity analysis model	2.32	0.56 – 9.62	0.246

*Adjusted for study variables as outlined in statistical analysis methods
† ID, Iron Deficiency
